# A theory of pulse dynamics and disturbance in ecology

**DOI:** 10.1002/ecy.2734

**Published:** 2019-05-20

**Authors:** Anke Jentsch, Peter White

**Affiliations:** ^1^ Disturbance Ecology Bayreuth Center of Ecology and Environmental Research BayCEER 95440 Bayreuth University Bayreuth Germany; ^2^ Biology University of North Carolina at Chapel Hill Chapel Hill North Carolina 27561 USA

**Keywords:** biodiversity, biogeography, biological traits, disturbance, ecosystem dynamics, resilience, stability

## Abstract

We propose four postulates as the minimum set of logical propositions necessary for a theory of pulse dynamics and disturbance in ecosystems: (1) resource dynamics characterizes the magnitude, rate, and duration of resource change caused by pulse events, including the continuing changes in resources that are the result of abiotic and biotic processes; (2) energy flux characterizes the energy flow that controls the variation in the rates of resource assimilation across ecosystems; (3) patch dynamics characterizes the distribution of resource patches over space and time, and the resulting patterns of biotic diversity, ecosystem structure, and cross‐scale feedbacks of pulses processes; and (4) biotic trait diversity characterizes the evolutionary responses to pulse dynamics and, in turn, the way trait diversity affects ecosystem dynamics during and after pulse events. We apply the four postulates to an important class of pulse events, biomass‐altering disturbances, and derive seven generalizations that predict disturbance magnitude, resource trajectory, rate of resource change, disturbance probability, biotic trait diversification at evolutionary scales, biotic diversity at ecological scales, and functional resilience. Ultimately, theory must define the variable combinations that result in dynamic stability, comprising resistance, recovery, and adaptation.

## Introduction

Pulse events, defined as abrupt changes in ecological parameters, are ubiquitous in ecosystems and include a wide array of phenomena, such as heat waves, marine upwelling, mass reproductive and mortality events, and biomass‐altering disturbances (Yang and Naeem 2008). Understanding pulse events is important because of this ubiquity and because the frequencies and magnitudes of such events as droughts, fires, floods, windstorms, and pest outbreaks, are changing because of human influences including, most importantly, climate change, land‐use change, and species invasions (Franklin et al. 2016, Seidl et al. 2017, Loehman et al. 2018, McDowell et al. 2018). In turn, pulse events can affect the responses of ecosystems to these influences, e.g., increasing invasion rates (Hobbs and Huenneke 1992) and accelerating or otherwise affecting responses to climate warming (DeFrenne et al. 2013). Characterizing pulse dynamics is also important as the basis for determining the degree of novelty of events (Hallett et al. 2013). Finally, event characteristics are central to understanding ecological resilience (Ratajczak et al. 2017), because pulse characteristics reflect ecosystem resistance, create the initial conditions for recovery, and are, by definition, the change to which the system may or may not be resilient (Carpenter et al. 2001). In this paper, we use a deductive approach to derive the minimum set of propositions, here called postulates (after Marquet et al. 2014), that create a general explanation for pulse dynamics across ecosystems and for places with different biogeographic histories. We then develop seven generalizations, phrased as predictions, for an important class of pulse events, biomass‐altering disturbances, that emerge from these postulates.

Our interest in a theory of pulse dynamics developed from the challenge of generalizing disturbance and ecosystems dynamics (White and Jentsch 2001). Disturbance ecology has produced a number of conceptual frameworks over the last several decades (Shugart 1984, White and Pickett 1985, Pulsford et al. 2016), including the intermediate disturbance hypothesis (Connell 1978, Fox 2013, Shiel and Burselm 2013), the dynamic equilibrium model (Huston 1979, 2014), the theory of nutrient dynamics (Vitousek and Reiners 1975, Vitousek 1984), the theory of forest dynamics (Shugart 1984), and the theory of landscape dynamics (Turner et al. 1993). Important recent frameworks include disturbance interactions and cross‐scale perspectives (Peters et al. 2004, Raffa et al. 2008, Buma and Wessman 2011, Buma 2015, Cannon et al. 2017), the concepts of biological legacy and ecological memory (Johnstone et al. 2016), generalizable biogeochemical responses to ecosystem disturbance (Kranabetter et al. 2016), the network‐based view on the role of disturbance in biodiversity and productivity (Gross 2016, Seidl et al. 2017), and patterns of trait diversity shaped by evolutionary trade‐offs (Diaz et al. 2016).

We build on this past work by proposing a general theory of pulse dynamics and disturbance that serves as an overall structure for the insights that have developed over the last several decades. This structure consists of a set of four fundamental postulates and the relationships among them that together circumscribe the common denominators and rules for all pulse events. Although disturbances can initiate successional change, we do not review theories of successional mechanisms and pathways here (see Glenn‐Lewin et al. 1992, Meiners et al. 2015, Peet 1992, Walker and del Moral 2003, Pickett et al. 2011, Walker and Wardle 2014, Pulsford et al. 2016); nor do we treat the dynamics among species that occur after pulse initiation, such as trophic interactions (see Holt 2008, Nowlin et al. 2008, Karakoç et al. 2018).

### Definition and types of pulse events

**Figure 1:**
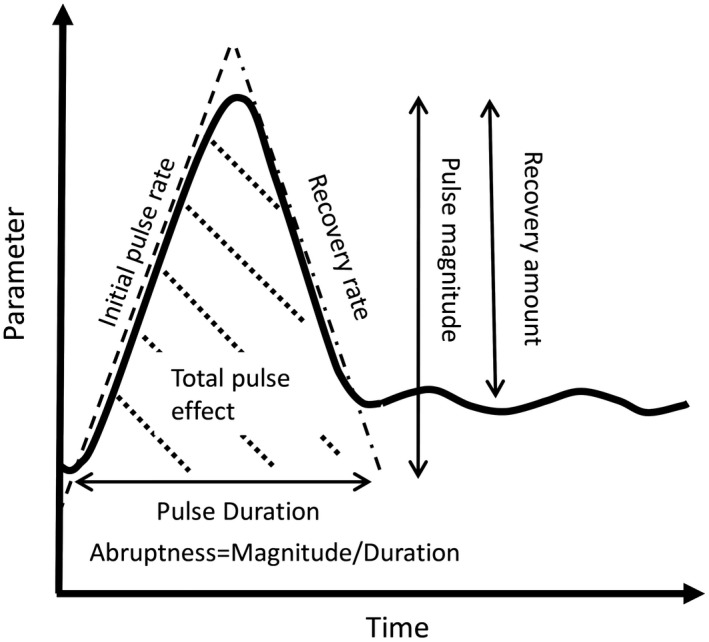
Quantifying the dimensions of pulse events. Seven variables that define pulse events: magnitude, duration, abruptness (magnitude/duration), initial rate of change, rate of recovery, magnitude of recovery, and the total pulse effect (area under the curve).

**Figure 2:**
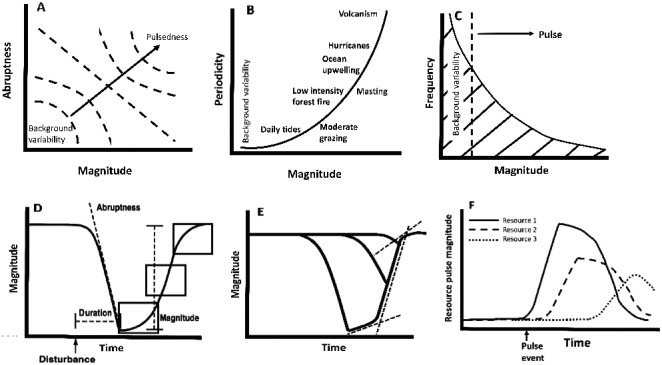
The characteristics that define pulse dynamics. (A) Pulse events vary by magnitude of change and abruptness. (B) Pulse regimes vary in periodicity, with the degree of variation in periodicity determining predictability. (C) The inverse relationship between magnitude and frequency of pulse events; the area below the curve is shaded because low‐magnitude pulses can occur at any frequency, but high‐magnitude pulses are generally constrained to low frequency (modified from White and Jentsch 2001). (D) The rate of change after pulse events (illustrated here by a high‐magnitude biomass‐altering disturbance) is initially limited by rate of colonization and organism response (lower box), and is finally limited by diminishing resources or space (upper box), with a maximum recovery rate at intermediate time since pulse initiation (middle box). (E) The initial rate of change (dashed lines) varies with pulse magnitude. (F) Pulse events initiate secondary pulses that can lead to synergisms such as feedback loops or cascades.

A pulse event is any abrupt change, positive or negative, in system parameters (Yang et al. 2008), with abruptness defined as the magnitude of the change divided by its duration (White and Jentsch 2001). Pulse events can be characterized by seven continuous variables that describe the dimensions of a particular parameter change: magnitude, duration, abruptness, initial pulse rate, the rate of recovery, the degree of recovery, and the area under the pulse curve (Fig. 1). Because magnitude and duration are continuous variables, pulse events become gradual changes as magnitude decreases and duration increases (sometimes called “presses”). This means that “pulsedness” (Yang et al. 2008) is, itself, a continuous variable (Fig. 2A, modified from Yang et al. 2008). However, we posit that resistance to pulse forces, discreteness of individual organisms, finite life spans, limits to niche breadth, and the interval between pulse events often result in thresholds and, therefore, discontinuities and patchiness. In essence, abruptness and discreteness can develop, at least at the scale of the individual, because event forces meet with biotic resistance that is ultimately limited. Similarly, the spatial propagation of some pulse events is characterized by thresholds leading to either low‐ or high‐magnitude events, for example, for disturbances such as insect outbreak and fire (Peters et al. 2004).

Disturbance has been defined both broadly and narrowly. In its broad sense, disturbance encompasses all pulse events (White and Pickett 1985). In its narrow sense, disturbance applies to a special class of pulse events characterized by direct alteration of biomass and ecosystem structure (Grime 1979), termed here “biomass‐altering disturbance” (see below). Under both definitions, the pulse perspective focuses on the dimensions of resource change and the mechanisms of response, whether a single disturbance event causes one resource pulse or a cascade of pulses over time.

We can recognize four types of pulse events (modified from Yang and Naeem 2008): (1) fluctuation in physical environmental conditions such as heat waves or droughts; (2) abiotic changes in resource supplies such as those caused by ocean upwelling or lake turnover; (3) changes in biotic resources through sudden demographic events, such as mass reproductive or mortality events (Holt 2008, Yang and Naeem 2008, Yang et al. 2008); and (4) changes through abrupt alteration of biotic structure, that is, biomass‐altering disturbances. Spatial subsidies, that is, the transfer of resources across space, have been recognized as a fifth category (Yang and Naeem 2008); however, these transfers involve forces such as wind, water flow, and gravity in the movement of resources, organic materials, soils, or geological substrates and thus can be considered forms of the second (abiotic changes in resource supply) or fourth (biomass‐altering disturbances) pulse types. Indeed, pulse events almost always result in the spatial movement of resources, though the scale of this movement varies from local to global. By including biomass‐altering disturbance within the larger framework of pulse dynamics, we explore the insight that disturbance can be generalized through the description of pulses of resource change, varying in direction, magnitude and rate, observable at a wide range of scales, and occurring at any trophic level.

**Figure 3:**
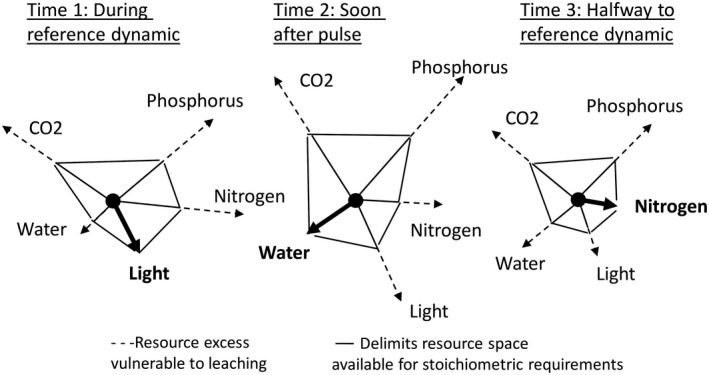
Resource stoichiometry and hierarchy in limiting factors in pulse dynamics. Lengths of arrows indicate resource amounts, from which resource ratios can be calculated, for example, in relation to the limiting resource. Time 1 = resource ratios during prepulse reference dynamics, Time 2 = resource ratios after the pulse event, Time 3 = resource ratios during return to prepulse conditions. Bold arrow shows the limiting resource at each time, which changes throughout recovery, for example, from light to water to nitrogen. Solid line shows the ratios among all resources. Dashed lines show the resources that are in excess (vulnerable to loss). Some resources change in a correlated pattern, others independently (not shown). Some resources remain static in amount but become limiting when other resources change.

Three scales are immediately apparent in pulse dynamics: the scale of the individual pulse event in time and space, the scale of multiple patches and events (i.e., the landscape scale, called the multipatch scale in White and Jentsch 2001), and the biogeographic scale (i.e., continent to global variation in environment and species pools). At the patch scale, the initial pulse sets off a sequence of further changes (Figs. 2 and 3) that are determined by abiotic and biotic processes, including changing ratios among resources and the transfer of resources to and from biotic and abiotic pools and across trophic levels (Bazzaz 1983, Bender et al. 1984, Holt 2008, Yang and Naeem 2008). At large scales, pulse dynamics are described by the spatial and temporal distributions of pulse events, such as size, dispersion, magnitude, frequency, and predictability (the “disturbance regime”; Fig. 2). The phrase “patch dynamics” has been used to describe ecosystem pattern and process at multiple scales in the disturbance ecology literature (Thompson 1978, White and Pickett 1985). Here, we use “pulse dynamics,” after Yang et al. (2008), to emphasize change in resources and environment. We retain the concept and phrase “patch dynamics” as the third of the four postulates to treat the spatial and temporal distribution of patches and events. The third scale in our treatment, the biogeographic scale, incorporates variation in environmental conditions and species pools. There are no fixed spatial or temporal dimensions for these three scales, but analytic scales can be derived in units relative to the size, dispersal characteristics, growth rates, and life spans of organisms, or from experimental and observational designs that allow analysis of the scale dependence of ecological responses.

## Challenges and Approaches to Writing Theory in Ecology

Approaches to the development of ecological theory are diverse (Pickett et al. 1994, Scheiner and Willig 2011, Vellend 2016). Here, we follow Marquet et al. (2014) by proposing a minimum set of essential postulates, defined as axiomatic law‐like statements and their underlying assumptions that constitute a general explanation (Marquet et al. 2015) of pulse dynamics. The resulting four postulates jointly explain the variation in pulse dynamics across ecosystems. In this sense, we seek theory based on first principles (Evans et al. 2013, Marquet et al. 2015), parsimonious in scientific rationale yet generating understanding and predictions (Ginzburg and Jensen 2004), explanatory and mechanistic (Mouquet et al. 2015), and deduced from logic rather than induced from empiricism (Huston 2014).

Our approach contrasts with attempts to create generality by synthesizing across empirical studies, an inductive approach that often leads to long lists of potentially important variables necessary to capture all the factors that are important in one or more ecosystems (e.g., White and Jentsch 2001, Peters et al. 2011). Vellend (2016) terms the elements of such lists “lower level factors” (i.e., climate, geography, soil/water properties, biotic interactions) and instead bases his theory of communities on four higher level processes: selection, drift, migration, speciation. In essence, our postulates seek to describe how Vellend's processes play out when parameters change abruptly. There will always be a dialog between deduction and induction, in the sense that empirical research relies, at least implicitly, on a logical structure that has determined what measurements should be made, whereas deduction is based on the empirical patterns and their potential causes.

The inevitable interplay between induction and deduction creates an understandable skepticism about the potential for theory in ecology. Here, we discuss four challenges to writing theory in ecology and our responses to them: (1) the dependence of current pattern and process on historical events and pathways, (2) the dependence of species adaptations on the evolutionary history of abiotic conditions and pulse events that embeds a potential circularity in explanations, (3) the intrinsic variation across ecosystems and species pools that is often missing from theoretical constructions, and (4) the inherent complexity in ecological phenomena. These challenges are, in part, responsible for a “continuing tension between logic and empiricism” (Huston 2014), such as in the debate over the intermediate disturbance hypothesis (Fox 2013; Shiel and Burselm 2013).

### Historical contingency at ecological scales

Historical events and pathways determine ecosystem states and species availabilities, and thus, current responses to pulse events are dependent on the spatial and temporal distribution of past pulse events (e.g., Raffa et al. 2008). The influence of ecological history on current processes is an example of path dependency in complex adaptive systems theory (Boero et al. 2014). As a result, current or initial conditions often have to be used to parameterize models in order to produce predictions, called “anticipatory predictions” or forecasts by Mouquet et al. (2015) in contrast to explanations or predictions from first principles that do not require historical data nor description of initial conditions. Here, we seek to create a structure that can generate the essential features of initial conditions and subsequent dynamics from first principles that encompass essential mechanisms.

### Dependence on evolutionary history

Dependence of species adaptations on the evolutionary history and thus on exposure to past environmental conditions means that the significance of a given environmental factor or degree of environmental change can be interpreted only through the responses of organisms. Ultimately, the separation of pulse from nonpulse dynamics and the significance of a given pulse event is based on organism response, including resistances and recovery rates. Thus, abiotic conditions and biotic responses constitute an adaptive system that has produced the tolerance and response traits of individuals for a give environmental change. Here, we address the interdependence of evolutionary responses and abiotic forces associated with pulse events by explicitly proposing that evolution has resulted in predictable patterns of trait diversity, complementarity, and redundancy under the constraints of phylogenetic descent and adaptive barriers (Scheffer et al. 2018, Postulate 4).

### Intrinsic variation among ecosystems and species pools

Theoretical constructs usually lack variables that represent the variability across ecosystems in function and species pools. For instance, Vellend (2016) notes that species and ecosystem type are usually treated as “givens” in ecological research. Here, we propose that ecosystem and species pool differences must be represented explicitly in theoretical terms. We propose that resource change can only be interpreted through and is constrained by stoichiometric requirements of organisms (Postulate 1), that differences in energy flow across ecosystems must be included explicitly in general theory because they control rates of ecological and evolutionary responses to pulse events (Postulate 2), that the scales of patch dynamics can only be interpreted through and are constrained by the range of dispersal ability and longevity of the biota (Postulate 3), and that the dispersion of species across trait space follows inherent evolutionary rules that are dependent on phylogenetic constraints and time (Postulate 4).

### Complexity in ecological phenomena

Four examples of the complexity inherent in ecosystems are responses are probabilistic, thereby limiting predictability; responses, even in simple cases, include indirect effects (e.g., a species response is not just dependent on pulse characteristics but also on the response of other species); a complete empirical characterization of all system details is impossible; and interactions among variables may be additive, multiplicative, or dependent on timing (and thus producing feedbacks). In this paper, our proposal is to define the role of theory as creating a mechanistic structure for models that incorporate stochasticity, indirect effects, uncertainty, and feedbacks.

## The Four Postulates Necessary for a Theory of Pulse Dynamics

We propose four postulates as a minimum set necessary to explain pulse dynamics and disturbance in ecology (Box 1, Fig. 3). Postulate 1 characterizes the magnitude, rate, and duration of abrupt resource change at the patch and event scale and underlies ecosystem differences. Postulate 2 characterizes energy flux across gradients, which controls resource accumulation rates at the patch scale. Postulate 3 characterizes the spatial and temporal distribution of pulse events, thereby integrating multiple patches and events. Postulate 4 characterizes the evolutionary responses to pulse dynamics and the way biotic trait diversity controls ecological response to pulse events. The theory is of pulse dynamics and disturbance combines the four postulates to produce a general structure for understanding ecosystem dynamics (Fig. 3).

Postulate 1: Resource dynamics. Pulse events initiate a series of predictable changes in resource ratios, storage and availability that are controlled by abiotic and biotic processes, including the stoichiometric requirements and resource accumulation rates of organisms.

Box 1

**The four postulates of the theory of pulse dynamics**

*Postulate 1: Resource dynamics*

Pulse events initiate a series of predictable changes in resource ratios, storage, and availability that are controlled by abiotic and biotic processes, including the stoichiometric requirements and resource accumulation rates of organisms.

*Postulate 2: Energy flux*

Energy flux determines the rate of ecological processes and responses to pulse events.

*Postulate 3: Patch dynamics*

The distribution of patches in space and time determines resources flows, variation in ecosystem structure, the availability of biota, and, thus, the nature of future pulse events.

*Postulate 4: Biotic trait diversity*

Pulse dynamics produce evolutionary forces that generate trade‐offs, leading to predictable patterns of trait diversification, and, in turn, the diversity, complementarity, and redundancy of biotic traits determine how ecosystems respond to pulse events.

**Figure 4:**
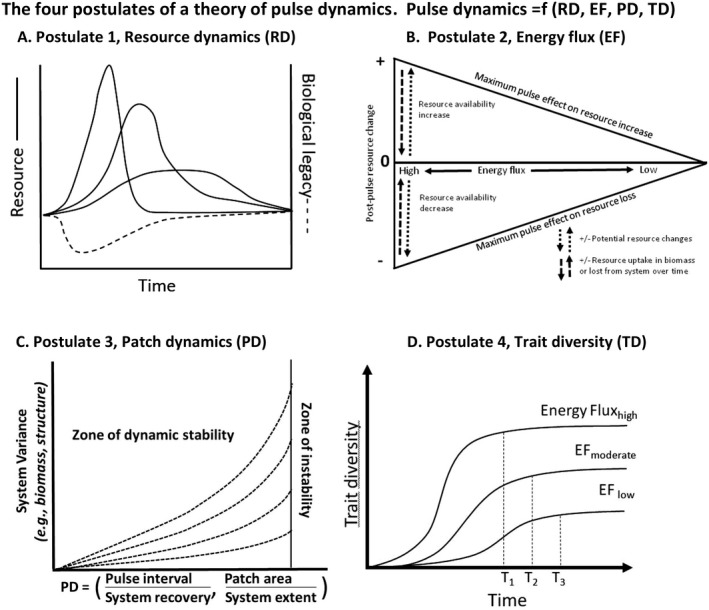
The four postulates of the theory of pulse dynamics. A. Postulate 1 (Resource Dynamics, RD) characterizes the magnitude, rate, and duration of abrupt resource change at the patch and event scale. B. Postulate 2 (Energy Flux, EF) characterizes variation in energy flux across gradients, which controls resource accumulation at the patch scale. Energy flux decreases from left to right, with the vertical axis representing potential effects of pulse events that can alternatively decrease (downward) or increase (upward) resource availability. The horizontal (zero) line depicts initial conditions. C. Postulate 3 (Patch Dynamics, PD) characterizes the spatial and temporal distribution of pulse events and the dependence of system variance and stability on two ratios, the ratio of pulse interval to system recovery time and the ratio of patch area to system extent (Turner et al. 1993). The dashed lines illustrate differences among ecosystems. The vertical line indicates that increasing variance can push systems beyond biotic tolerances, so that instability occurs (species and pathways of change are lost). D. Postulate 4 (Trait Diversification, TD) characterizes the evolutionary responses to pulse dynamic. We assume that the asymptote and rate of saturation both increase with energy flux, leading to increasing time to saturation (T_1_ > T_2_ > T_3_). All four postulates interact in creating the phenomenon of pulse dynamics.

As essential assumption of Postulate 1 is that there are predictable and generalizable pathways of changes in resource availability, where resource is defined as any aspect of an organism's environment that it uses to increase its own fitness while pre‐empting other organisms from using that same resource. The pathways of resource dynamics depend on the magnitude of the pulse event (Iwasaki and Noda 2018), on rates of ecosystem processes based on resource flux (Postulate 2), and on the processes of biotic uptake, determined by the availability of organisms, their stoichiometric requirements, and their physiological states (Vitousek 1984, Sterner and Elser 2002, Rastetter et al. 2013, Helton et al. 2015, van Huysen et al. 2016; Fig. 2). Because the availability of multiple resources is affected by one event, resource ratios change and alter the nature of the limiting resource for biotic processes (Fig. 4). Stoichiometric requirements of organisms also determine the resource pools contained in biomass, and, hence, the potential resource changes that result from pulse events that alter biomass. Species differ not only in rates of uptake of resources but also in the resources they accumulate in biomass and the ecosystems structures that result.

The pulse events that initiate resource dynamics can also change the availability and functional performance of the biota, thereby influencing resource availability through altered uptake rates rather than resource supply. For instance, abrupt changes in environmental characteristics may cause mortality but can also affect resource supply by altering the physiological states and resource uptake rates of organisms.

Postulate 2: Energy flux. Energy flux determines the rate of ecological processes and responses to pulse events.

An essential assumption of Postulate 2 is that ecosystems can be characterized by variation in energy flux. Postulate 2 also assumes that variation in energy flow and resource availability (Postulate 1) jointly drive resource uptake and dynamics (Margalef 1975, Brown et al. 2004). The magnitude of the resource change (Postulate 1) and energy flux (Postulate 2) determine the pressure for rapid colonization, when resources are abundant after the initiation of a pulse event (Postulate 4).

Postulate 3: Patch dynamics. The distribution of patches in space and time determines resources flows, variation in ecosystem structure, the availability of biota, and, thus, the nature of future pulse events.

Underlying Postulate 3 are three assumptions that describe the interrelationships of patch dynamics and biodiversity. First, patch dynamics produce dynamic behavior at large scales, including pulse interactions and feedbacks (Raffa et al. 2008). Second, patch distribution in space and time determines the flows of resources and the availability and responses of species, thereby making pulse history important to current response, influencing the role of pulse events in ecosystem resistance and recovery (Turner et al. 1993, Isbell et al. 2015). Third, species life histories, including life span, growth rates, and colonization rates, are constraints that determine the ecological significance of spatial and temporal characteristics of pulse events.

Postulate 4: Biotic trait diversity. Pulse dynamics produce evolutionary forces that generate trade‐offs, leading to predictable patterns of trait diversification, and, in turn, the diversity, complementarity, and redundancy of biotic traits determine how ecosystems respond to pulse events.

**Figure 5:**
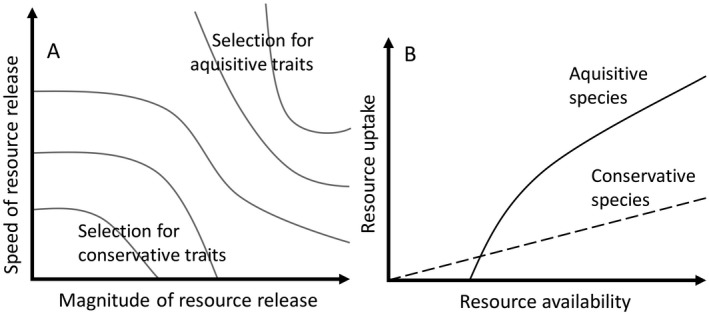
Speed of resource release, magnitude of resource release, and energy flux predict the strength of selection for biotic trait diversification with regard to ecological trade‐offs. (A) Two axes, speed and magnitude, define the abruptness of resource change and create the selection pressures as a function the trade‐off between acquisitive‐conservative strategies. (B) Resource uptake rate increases with resource availability for both acquisitive and conservative strategists, but the acquisitive traits lead to higher resource uptake at high resource availability.

Underlying Postulate 4 are two assumptions about the evolution of biotic trait diversity. First, niche diversification and trait evolution are saturating, time‐dependent processes, with evolutionary rate positively correlated with energy flux and negatively correlated with accumulating diversity (Fig. 5, Walker and Valentine 1984, Kraft and Ackerly 2010, Hurlbert and Stegen 2014, Swenson et al. 2016). Ultimately, energy flow and resource availability (Postulate 1) jointly drive resource uptake and dynamics (Margalef 1975, Brown et al. 2004) and have been hypothesized to predict evolutionary rates (e.g., the energy‐diversity theory, Hurlbert and Stegen 2014). The second assumption of Postulate 4 is that the mechanisms of trait evolution in the context of pulse dynamics are based on universal trade‐offs (Diaz et al. 2016), including the “acquisitive vs. conservative” resource capture strategy, the “colonization vs. competition” strategy, the “avoidance vs. tolerance and resistance” strategy, the “specialist vs. generalist” strategy, and the “reproductive output vs. longevity” strategy (Yang et al. 2008, Diaz et al. 2016). Whether implicitly or explicitly, all generalizations about pulse dynamics (e.g., the intermediate disturbance hypothesis, as discussed below) require trait differentiation based on trade‐offs. Quantifying the distribution of species in trait or niche space, and testing the assumption that trait diversification is a saturating and predictable process is an ongoing research question (Winemiller et al. 2015, Bruelheide et al. 2018).

The evolutionary pressures expressed through the trade‐off between colonization and competition is a distinctive feature of those pulse events that increase resource availability: the greater the magnitude and abruptness of the resource pulse (Postulate 1) and the greater the energy flux (Postulate 2), the higher the pressure for fast colonization and resource uptake. The abruptness of pulse events also allows produced stochastic influences (drift in Vellend's sense) to be more important than they are with static spatial gradients, where competition (selection in Vellend's sense) may be the driving species sorting.

The assumption that universal trade‐offs produce generalizable patterns in trait diversity essentially embeds an argument for adaptive efficiency (Margalef 1975, Jørgensen et al. 2007) that maximizes the components of fitness per unit of energy flux (Lotka 1922a, b). Thus, energy flux ultimately can link evolution with metabolism (Brown et al. 2004), because fitness can be expressed in units relative to energy use (Lotka 1922a, b). A central premise of systems ecology is that energy flow is used to create order and information and thus necessarily also produces entropy as a consequence of the second law of thermodynamics (Fath et al. 2004, Jørgensen and Fath 2004, Chapman et al. 2016).

There are two important consequences of the assumption of universal trade‐offs: first, trait diversification is correlated with an increase in resource use efficiency, a principle termed the “maximum power principle” in the systems ecology literature (Odum and Pinkerton 1955, Chapman et al. 2016), although the potential increase in efficiency is determined by genetic diversity and phylogenetic, biological, and physical constraints, and, second, trade‐offs result in increased functional complementarity and redundancy among organisms, both of which contribute to ecosystem response to pulse events. Thus, pulse dynamics are one cause of an increasingly strong correlation between biodiversity and ecosystem function as temporal and spatial scale increases (Oliver et al. 2015).

The evolutionary pressures generated by pulse events have also been described for resource–consumer interactions and have been tied to four consumer strategies that affect species abundances and stability (summarized from Ostfeld and Keesing 2000, Holt 2008, Yang et al. 2008). First, “spatial averaging” means that mobile consumer populations search for and integrate resource supply over heterogeneous landscapes such that the spatially shifting and ephemeral patches provide a stable resource supply for the consumer at a larger scale. Second, “generalist behaviors” includes diet switching, which lessens the relationship between consumer and the pulse availability of any one resource. Third, the “tolerator strategy” allows consumers to persist during times of low resource availability, e.g., through dormancy. Fourth, the “aggregative response” describes how quickly consumer populations colonize an ephemeral patch. Pulse events propagate through time and trophic webs via resource or demographic storage, indirect effects, extinction or invasion, and genetic changes (Yang et al. 2008).

## The Four Postulates Applied to Biomass‐Altering Disturbances

Biomass‐altering disturbances (disturbance in the narrow sense of the term, Grime 1979) are an important class of pulse events that is distinguished by three linkages between pulse forces and ecosystem responses (Fig. 2). First, ecosystem structure and biotic traits create ecosystem resistance to a disturbance of a given intensity (Pickett and White 1985). Second, the resources stored in living and dead organic structures are linked to changes in resource availability after disturbance. Finally, the legacy of the predisturbance ecosystem influences resource uptake rates after disturbance (James et al. 2007, Johnstone et al. 2016). Below, we apply the four postulates to disturbance events and discuss research needed for the further development of a theory of disturbance (Table 1).

**Table 1:** Assumptions and embedded functions of the postulates and predictions that need further specification in the application and test of a general theory of pulse dynamics and disturbance in ecology

Postulate(s)	Assumption/embedded functions
RD	The sequence of resource changes after a given pulse event is a function of the stoichiometric requirements of organisms constrained by energy flux (under the assumption of the evolution of resource use efficiency).
EF	Energy flux can be quantified as a function of environmental conditions and resources, producing a continuous variable that controls ecosystem responses to disturbance events.
PD	Patch distribution in space and time, relative to species traits (e.g., longevity, reproductive period, dispersal) determines thresholds for persistence of species and biotic trait diversity.
TD	Rate of trait diversification is a function of energy flux, the magnitude and duration of resource pulses, and, through feedback, existing trait diversity.
TD	The form of the distribution of species in trait space results from a saturating evolutionary process determined by energy flux, resource availability, and time.
RD, EF, TD	Pattern and rate of resource accumulation is a function of energy flux, resource availability, and niche characteristics (breadth, redundancy, and complementarity for resource acquisition and disturbance resistance), leading to generalizable patterns of biomass and resources contained in biomass over time.
PD, TD	Resilience results from the spatial and temporal distribution of resource pulses and biotic trait diversity.

Notes

Postulates: RD = resource dynamics, EF = energy flux, PD = patch dynamics, TD = biotic trait diversity.

### Resource dynamics in disturbance ecology

In biomass‐altering disturbances resource availability changes as a function of the force acting on biomass (Iwasaki and Noda 2018). Resource availability after disturbance changes in four ways: through transformation of resources directly by the disturbance event; through changes in biotic uptake; through flows of organic matter, nutrients, water, and atmospheric gases; and through release of resources stored in biomass. Resource ratios also change, thus altering which resources are limiting to organisms (Fig. 4). Resources that are not limiting may be lost through leaching or abiotic transformation (Vitousek and Reiners 1975), although abiotic immobilization and luxury consumption can also occur (Chapin 1980). Biogeochemical transformations depend on energy pathways (Helton et al. 2015; Postulate 2). Producing a general model of the effects of disturbance on biogeochemistry is a major, but unfinished task that is required for generalizable theory (Table 1; Kranabetter et al. 2016).

Although resource storage and biomass generally increase over succession, long‐term succession in the absence of disturbance can result in a retrogressive phase defined by decreasing resource availability and storage in biomass (Walker and Wardle 2014). Change in resource availability after disturbance then depends on the timing of disturbance relative to succession, an illustration of the link between resources in biomass and potential changes in resource availability caused by disturbance (Peltzer et al. 2010, Walker and Wardle 2014).

### Energy flux in disturbance ecology

Energy flux determines the rate and pattern of resource accumulation with time since disturbance. Linking energy flux to resource dynamics is a requirement for development of a theory that can be applied across ecosystems and local or global gradients. One of the challenges is thus to define a metric for energy flux (Table 1). A proposed metric in the systems ecology literature is “entropic output.” Skene (2013) defined entropic output as the energy released as heat during carbon fixation and during the development of ecosystem structure over successional time.

For primary producers, energy flux is manifest as gross productivity, whether the assimilated energy is used for storage, ecosystem structure, defense, respiration, or reproduction. For example, photosynthetic capacity (the maximum rate of carbon fixation) has been modeled using radiation, temperature, humidity, and photoperiod (Ali et al. 2015). Net primary production has been modeled using growing season length (Chu et al. 2015). Net carbon storage has often been used as a proxy for energy. Gross primary production has been modeled using radiation, carbon dioxide concentration, temperature, moisture, seasonality and inorganic resources, or their proxies, plus leaf area index and canopy height (Williams et al. 1997, Enquist et al. 2003). The difference between standing and potential biomass across the globe is determined by time since biomass‐altering disturbance (Midgley and Niklas 2004).

### Patch dynamics in disturbance ecology

The theory of landscape dynamics (Turner et al. 1993) predicts ecosystem dynamics from two spatio‐temporal ratios: (1) the ratio of disturbance area to landscape area and (2) the ratio of disturbance return interval to recovery time. Equilibrium landscapes, with low to high variance, are predicted when disturbed patches are smaller than a critical fraction of landscape area and disturbances intervals are greater than recovery time, so that patches fully recover between disturbances (Turner et al. 1993). The theory of pulse dynamics generalizes this model of landscape dynamics in three ways: first, by using disturbance magnitude as change in resources rather than patch area alone (Postulate 1); second, by allowing for differences in rates of recovery based on differences in energy flux (Postulate 2); and, third, by predicting the array of biotic traits that have evolved in response to pulse dynamics (Postulate 4).

The Turner et al. (1993) theory predicts that stable landscapes can exhibit low to high variance. This characteristic variance has been proposed as a signature of disturbance events that is useful in monitoring and in comparisons among ecosystems and disturbance regimes (Fig. 3; Fraterrigo and Rusak 2008, Ratajczak et al. 2017). Stability with variance also underlies the concepts of the historic range of variation and qualitative (or persistence) equilibrium (reviewed in White and Jentsch 2001), and is important in the definition of ecosystem resistance and resilience.

The longevity, reproductive period, and dispersal characteristics of species relative to the timing and spatial dispersion of patches determine species abundances (as discussed from early on in Paine et al. 1998) and how species respond in disturbed landscapes that have varying degrees of connectivity (Shackeford et al. 2016). This produces two temporal and spatial contingencies: the evolutionary exposure to disturbance events in the past influences biotic traits, and the recent ecological history of disturbance determines whether species are present (e.g., for plants, reproductive and within dispersal distances or in the seedbank) and can then respond to current disturbances. Thus, patch dynamics create the biological legacies and the spatial and temporal variations in ecosystem composition and structure that control the availability of species and the functional traits for response to disturbance events. Further, the ecological significance of the dimensions of disturbance events in space (i.e., patch size and dispersion) and frequency in time (i.e., return interval) is determined by the life history traits of the biota present.

The spatial and temporal distribution of disturbance events and the biotic traits interact to predict patterns of diversity. Although the intermediate disturbance hypothesis (IDH, Connell 1978) and the dynamic equilibrium model (DEM, Huston 1979, 2014) were phrased as mechanisms of coexistence at the community scale, the theory of pulse dynamics predicts coexistence and a unimodal richness relationship at scales large enough to encompass all patch types and under conditions in which patches are neither so rare in time nor distant in space that they exceed the constraints imposed by species longevities and dispersal distances (Mouillot et al. 2013). Both IDH and DEM require trait diversity under evolutionary trade‐offs (Postulate 4). Huston's (1979, 2014) dynamic equilibrium model predicts diversity from the joint effects of disturbance—represented as the rate of mortality—and productivity. Here, we use resource availability (Postulate 1) and energy flux (Postulate 2) rather than productivity.

Given human influences on dynamic processes such as fire, flood, and pest outbreaks, patch dynamics is a major factor in species abundances, including vulnerability to extinction. These disturbance events also interact with human land‐use patterns and habitat fragmentation (Shackeford et al. 2016). Hence, understanding the importance of patch dynamics and life‐history constraints in species persistence is a fundamental issue in ecosystem management and conservation.

### Biotic trait diversity in disturbance ecology

Disturbances create evolutionary pressures in three ways: first, selection for traits that produce sensitivity vs. resistance to disturbance forces; second, selection for traits that produce rapid colonization vs. fast resource accumulation after disturbance; and third, selection for traits that alter the frequency and magnitude of disturbance events. Some species affect disturbance regimes and biodiversity as ecosystem engineers by changing landscape‐level processes, including the promotion or delay of future disturbances (Hobbs et al. 2006, 2009). For instance, species with highly flammable litter promote fire and species with the ability to fix nitrogen alter ecosystem nutrient fluxes (Ayanu et al. 2015, Vetter et al. 2018).

We propose that the evolutionary pressure for rapid colonization is a product of the magnitude and abruptness of resource increase (Postulate 1) and energy flux (Postulate 2) (Fig. 5) and therefore that differentiation of species along a colonization‐competition trade‐off is strongest at high energy flux so successional roles become more differentiated as energy flux increases (Postulate 2) along gradients from low‐ to high‐productivity ecosystems (Fig. 3).

Though succession has been described as a gradient in time, in analogy to gradients in space (see discussion in Pickett 1976), temporal gradients produced by disturbances differ in that they initially select for rapid arrival and establishment vs. selecting for traits that are the most efficient under long‐term resource competition. We can extend this contrast to spatial gradients that are dynamic under climate change but lack biomass‐altering disturbances. Climate change shifts species performance along spatial gradients, but the underlying model remains one of competitive replacements driven by resource use efficiency, although the existing ecosystem can have an intertial effect with resulting time lags in response to climate change. Biomass‐altering disturbances can remove this inertial effect, but they also select for species with rapid colonization and establishment rates that are accompanied by shorter life spans and the less efficient resource use that leads to competitive replacement during succession.

## Predicting Disturbance Dynamics

We combine the four postulates to produce seven generalizations that predict dynamics caused by biomass‐altering disturbances (Table 2). Predictions 1–3 address disturbance‐induced resource dynamics; prediction 4 addresses successional feedbacks and disturbance interactions; predictions 5 and 6 address species and trait diversity; and prediction 7 addresses resilience and regime shift.

**Table 2:** Seven predictions for biomass‐altering disturbances derived from the four postulates

Title	Prediction	Symbolic math	Examples of metrics and proxies
Resource dynamics			
1 Disturbance magnitude	The magnitude of a resource pulse (Postulate 1) is a product of the force of the disturbance and the accumulation of resources in biomass (Postulate 2) and biotic resistance to the disturbance (Postulate 4).	R_released_ = F_event_ × R_stored_ × (1−TD_resist_), where R_stored_ = AR_*t*_ × time R = resources, F = force, AR = resource accumulation rate, *t* = time, TD_suscept_ = relative amount of resistant trait diversity	Amount of biomass converted, i.e., from living to dead, tissue damage. Change in resource ratios, e.g., C:N:P ratios in soil and leaf chemistry. Change in physical environment, e.g., light, soil water, plant available nitrogen.
2 Resource trajectory	Resource trajectories and ratios after the initial pulse (Postulate 1)—including the changing hierarchy of the limiting factors—are a function of biotic stoichiometric requirements of the biota present (Postulate 4), and energy flux constraints (Postulate 2).	(RD_*x*_:RD_*y*_)_*t*_ = (RD_*x*_ − SR_*x*_)_*t*0_ : (RD_*y*_ − SR_*y*_)_*t*0_ RD = resource dynamics, SR = stoichiometric requirements, *t* = time.	Repeated measures of ecosystem resources, e.g., substrate chemistry vs. leaf chemistry. Accumulation of soil organic matter and aboveground biomass, e.g., canopy cover and leaf area.
3 Rate of change	The rate of change in resources and the turnover of species and traits after pulse events is a product of resource availability (Postulate 1) as determined by disturbance magnitude and energy flux (Postulate 2), of biological legacy (Postulates 1 + 3), and of biotic traits (Postulate 4).	AR = EF × R_released_ × TD R = resources, EF = energy flux, AR = resource accumulation rate, TD = biotic trait diversity.	Rate of change calculated from repeated measures of, e.g., resources, soil organic matter, cover, biomass, species richness, and biotic trait diversity.
Feedback and interaction			
4 Disturbance probability	In pulse dynamics, which are driven by ecosystem feedback, the probability of the next disturbance is a function of time since disturbance (Postulate 1 + 2).	D_prob_ = ES_*t*_/ES_max_ D = disturbance, ES = ecosystem state, *t* = time. The form of this function can be linear, exponential, or depending on critical thresholds.	Probability of disturbance as a function of time since disturbance measured as, e.g., changing preconditions for the next event, such as biomass‐related wind and drought susceptibility, age related to insect infestation, fuel load and fuel connectivity related to fire propagation.
Biodiversity			
5 Biotic trait diversification	Trait diversity increases in a saturating manner as a function of resource heterogeneity in space and time (Postulate 1 + 3), energy flux (Postulate 2), and biotic trade‐offs (Postulate 4), leading to a predictable pattern in species pool differentiation across trait space.	TD_t_ = TD_max_/{1 + [(TD_max_ −TD_*i*_)/TD_*i*_ ] × *e* ^−TDrate × *t*^} TD_*t*_ = biotic trait diversity, max = maximum, *i* = initial, TD_rate_ = trait diversification rate TD_max,_ TD_rate_ = *f*(EF, pulse magnitude)	Functional trait diversity, e.g., specific leaf area, seed weight, growth height, leaf nutrient status, specific stem density. Frequency distribution of species, such as trees, across trait values, such as spectrum of specific leaf area. Frequency distribution of traits, such as growth height, across ecosystems, such as spectrum of high‐elevation grasslands.
6 Species and trait diversity	Species and biotic trait diversity in ecological time are a function of evolutionary biotic trait differentiation (Postulate 4), patch dynamics (Postulate 3), energy flux (Postulate 2), and resource heterogeneity (Postulate 1).	TD_existing_ = TD × PD × EF × RD SD_existing_ = TD × PD × EF × RD SD = species diversity TD = biotic trait diversity as a result of evolutionary processes, PD = spatio‐temporal arrangement of patch dynamics, EF = energy flux, RD = resources	Species richness. Diversity indices, e.g., Shannon‐Weiner index, Simpson index. Beta‐diversity indices, e.g., Bray‐Curtis similarity index. Functional trait diversity measures, e.g., Rhao's Q index.
Resilience			
7 Functional resilience	Resilience is a function of biotic trait diversity (Postulate 4) in relation to resource availability (Postulate 1), energy flux (Postulate 2), and the spatial and temporal distribution of patches (Postulate 3).	RS = TD_existing_/TD_saturated_ × R × EF. RS = resilience, TD = biotic trait diversity, R = resources, which have been made available by the pulse, EF = energy flux, which is a function of resource accumulation rate.	Biotic trait diversity, such as redundancy and complementarity within a community and asynchrony among species. Degree of return to the reference level and time to new equilibrium state. Rate of change to the new equilibrium state. Cumulative magnitude of functional variation. Magnitude of initial pulse effect, e.g., resistance.

*Prediction 1—Disturbance magnitude* (Table 2): Disturbance magnitude, expressed as changes in resource availability, resource ratios, limiting resources, and biotic legacy, are the product of the force (or intensity) of the disturbance and ecosystem resistance (Iwasaki and Noda 2018).
*Prediction 2—Resource trajectory* (Table 2): Resource trajectories after disturbance are determined by the stoichiometric requirements of organisms, energetic constraints, physiological capacity, luxury uptake, and loss through spatial transfers (e.g., via water, wind, and gravity). Resource uptake changes the hierarchy of limiting.
*Prediction 3—Rate of change* (Table 2): Energy flux, resource availability, and biotic traits determine the rate of resource change after disturbance. In primary successions characterized by no biological legacy and multiple limiting resources, rates of change are slow at first, increase to a maximum, and then decrease again as resources are accumulated in biomass or lost through biotic processes, abiotic processes, or export. In secondary successions, rates of change are initially high and similarly decrease through time as available resources are accumulated in biomass or are lost from circulation.
*Prediction 4—Disturbance probability* (Table 2): Feedbacks between ecosystem structure and disturbance frequency change the probability and magnitude of future disturbance events as a function of time since disturbance and the legacy of those past disturbances. A major consequence is that feedback and disturbance interactions can increase or decrease the probability of future disturbances and can lead to cascading disturbances (Frelich and Reich 1999, White and Jentsch 2001, Allen 2007, Raffa et al. 2008, Buma 2015, Seidl et al. 2017). Examples of ecosystem feedback include increasing fuel levels through time, which increases fire risk. Examples of disturbance interactions include droughts that increase susceptibility to insect infestations, or flooding, landslides, and human fragmentation that reduce fuel connectivity and thus fire occurrence. A case of special interest in disturbance ecology occurs when, for systems and processes under human management (e.g., fire and flood), the decrease in disturbance frequency due to suppression leads to rarer but higher‐magnitude disturbances (the suppression hypothesis).
*Prediction 5—Biotic trait diversification in evolutionary time* (Table 2): Trait diversification is a saturating, evolutionary process that results in predictable trait distributions for such traits as dispersal, resource accumulation, longevity, and competitive interaction. The evolutionary pressure for trait diversification increases with energy flux, resource availability, abruptness of change, and resource heterogeneity.
*Prediction 6—Species and trait diversity in ecological time* (Table 2): Species and trait diversity at the landscape scale increase with biotic trait differentiation over time (Postulate 4), patch distribution in time and space (Postulate 3), energy flux (Postulate 2),and resource heterogeneity (Postulate 1).
*Prediction 7—Resilience* (Table 2): Resilience, defined as recovery after pulse events (Hodgson et al. 2015, Oliver et al. 2015, Donohue et al. 2016, Willis et al. 2018), can be quantified by the degree of return to reference level, rate of return, and time for reaching the former or a new steady state (Hodgson et al. 2015, Isbell et al. 2015, Todman et al. 2016, Feng et al. 2017, Craven et al. 2018, Ingrisch and Bahn 2018). Resilience is a function of disturbance magnitude and frequency relative to trait diversity under the assumption that the higher the biotic trait differentiation, the greater the asynchrony, redundancy, and complementarity of traits and thus the capacity for functional recovery (Oliver et al. 2015).

## Further Development of a Theory of Pulse Dynamics and Disturbance

Deduction and empiricism are complementary approaches in the development of ecological theory. We have taken a deductive approach in creating a broadly explanatory framework based on a minimum set of principles that captures the fundamental variables and the relations between them. In the disturbance literature, syntheses and modeling across field studies has been an empirical approach to generalization (e.g., White and Jentsch 2001, Peters et al. 2011). Whether deductively or empirically derived, theories present frameworks that specify the factors and relationships deemed sufficient for general explanation. However, there is another level to theory—theory that predicts particular cases defined by the parameter space. For pulse dynamics, the most important focus of theory is the prediction of conditions for stable and unstable ecosystem dynamics.

Understanding pulse dynamics is an essential part of all resilience theories because these dynamics produce the response of the system to pulse events (Carpenter et al. 2001, Ingrisch and Bahn 2018). Within the array of dynamic possibilities produced by the four postulates, theory must seek to define the variable combinations that result in mechanisms of stability, comprising resistance, recovery (resilience in the narrow sense of that term), and adaptation, vs. those conditions that create unstable dynamics and regime shifts (e.g., Ratajczak et al. 2017). Ultimately, biotic diversity, whether represented by traits, species, niche complementarity and redundancy, or genotypes and phenotypic variation, underlies resistance, recovery, and adaptive possibilities in pulse dynamics.

Progress on a quantitative theory of pulse dynamics and disturbance in ecology will come from mathematical expression of the postulates (Table 1), especially for generalizable pathways of resource transformation under energetic and stoichiometric constraints (Postulates 1 and 2) and generalizable patterns of trait evolution and interactions among organisms (Postulate 4). This development will allow testing these ideas in a broad array of biogeographical settings and across the inherent variability in pulse events and regimes.
